# Supplementary material for: Asymmetrically Substituted Phospholes as Ligands for Coinage Metal Complexes
Source: Molecules. 2022 May 24;27(11):3368. doi: 10.3390/molecules27113368 (PMC9182544; doi:10.3390/molecules27113368)
Supplement: Supplementary file 1 [file molecules-27-03368-s001.zip › molecules-1724150-supplementary.pdf]

Supporting Information

Asymmetrically substituted phospholes as ligands for coinage metal complexes

Fabian Roesler, Clemens Bruhn and Rudolf Pietschnig*

Table of Contents

NMR spectra of compound **3**	3
NMR spectra of compound **4**	4
NMR spectra of compound **5**	6
^{1}H DOSY-ECC-MW estimation of **3**	8
^{1}H DOSY-ECC-MW estimation of **4**	9
^{1}H DOSY-ECC-MW estimation of **5**	10
Crystallographic data	11
Ring numbering scheme in phospholes	13
References	14

NMR spectra for compound **3**

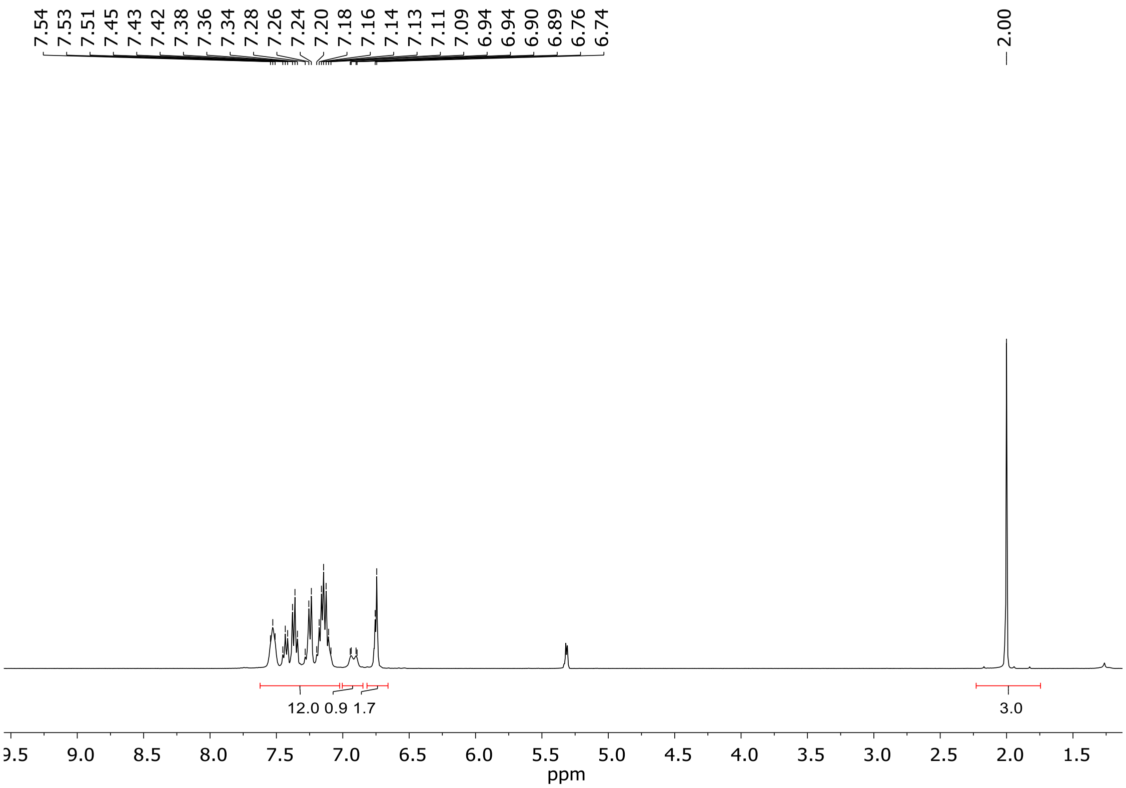

Figure S1: ^{1}H NMR (CD_2Cl_2, 400 MHz).

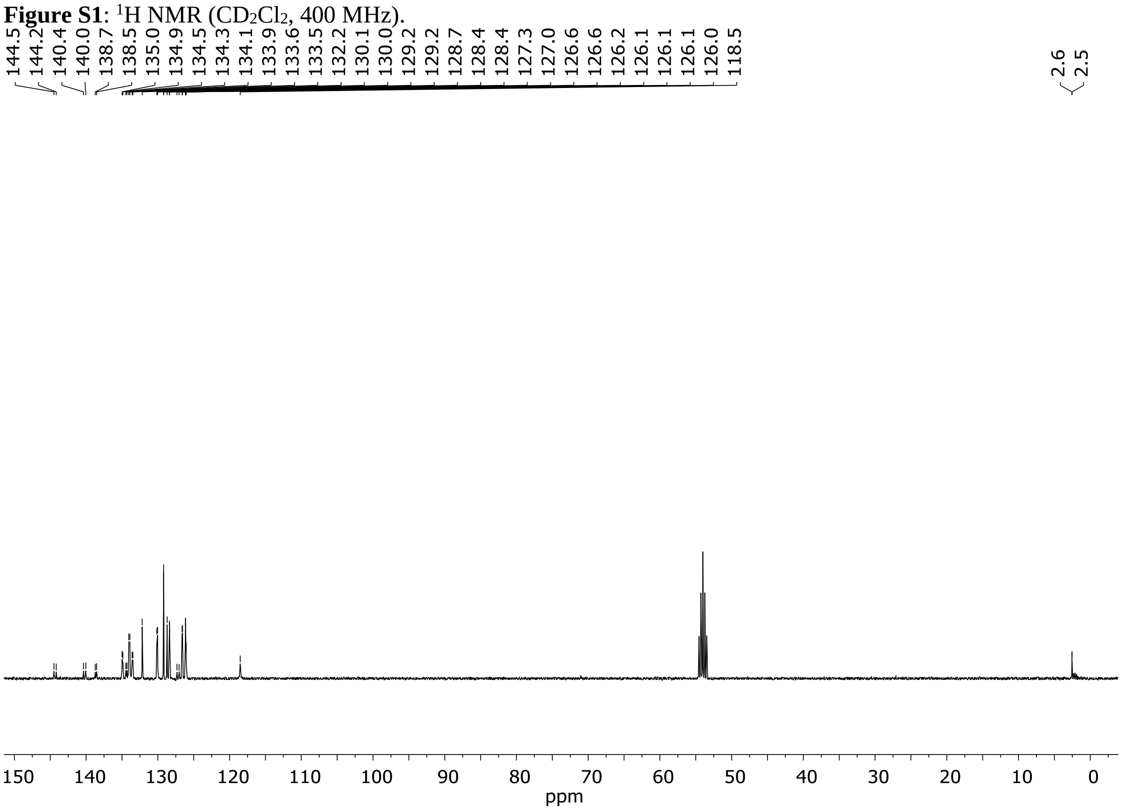

Figure S2: ^{13}C NMR (CD_2Cl_2, 101 MHz).

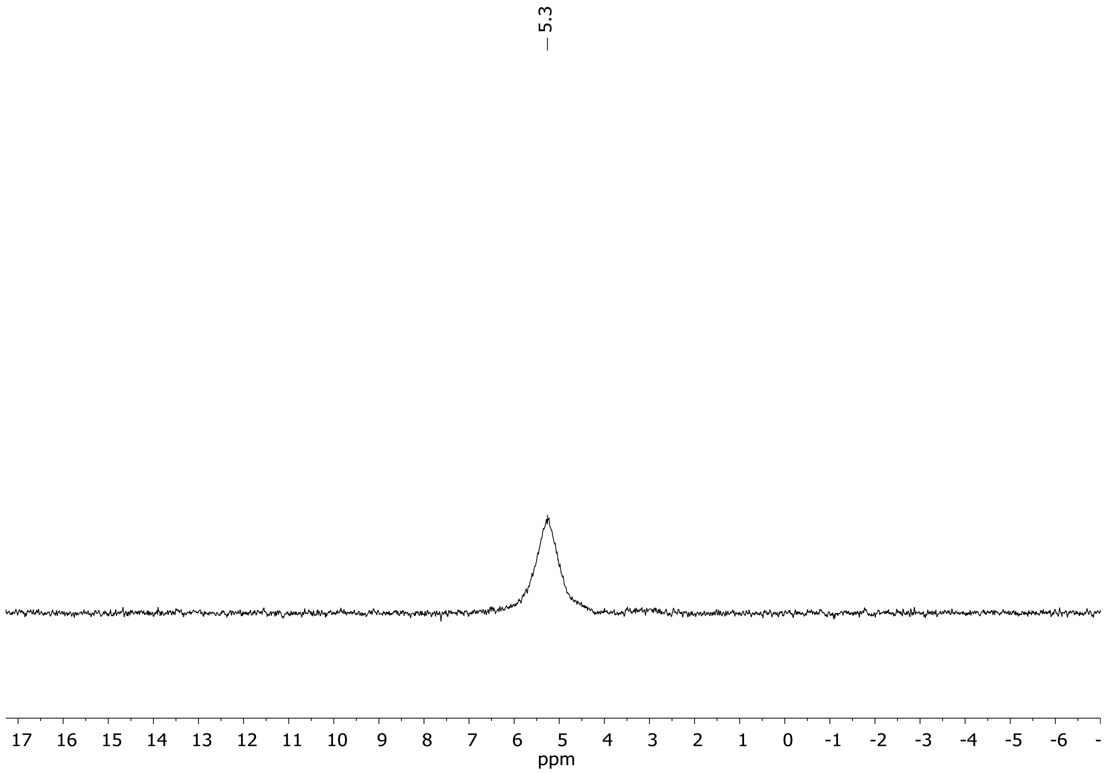

Figure S3: $^{31}P\{^{1}H\}$ NMR (CD_2Cl_2, 202 MHz).

NMR spectra for compound **4**

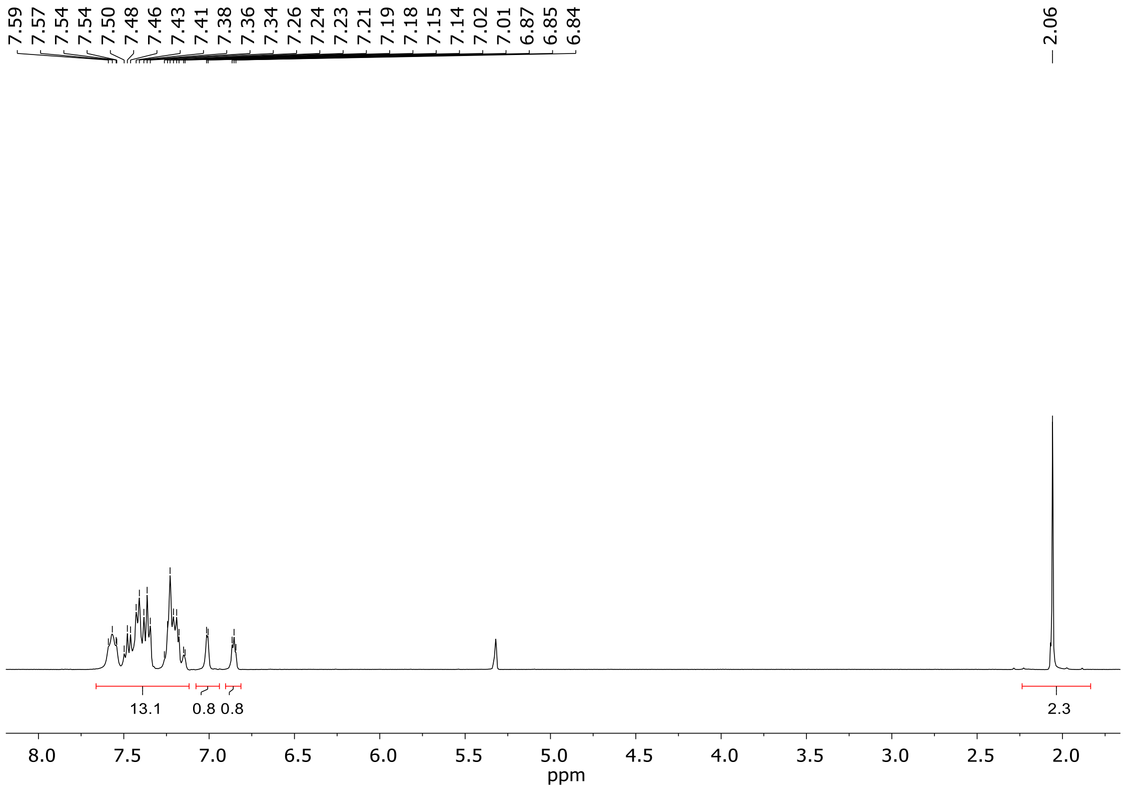

Figure S4: ^{1}H NMR (CD_2Cl_2, 400 MHz).

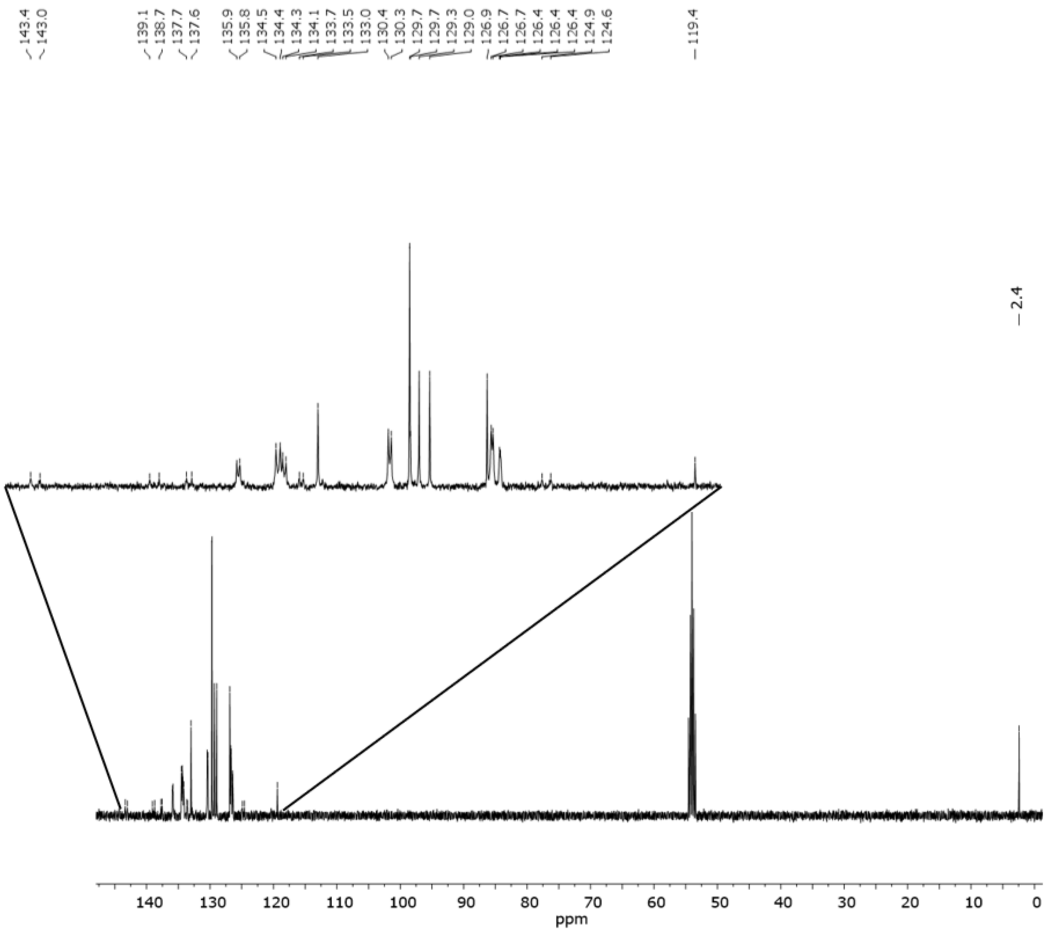

Figure S5: ^{13}C NMR (CD_2Cl_2, 101 MHz).

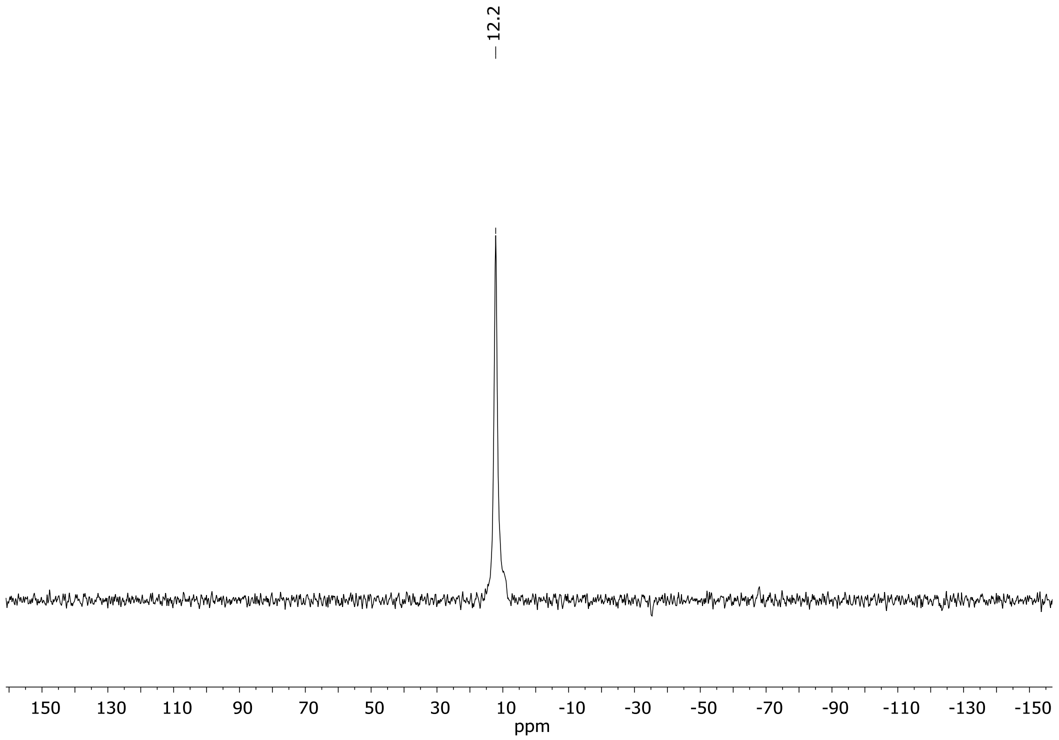

Figure S6: $^{31}P\{^1H\}$ NMR (CD_2Cl_2, 202 MHz).

NMR spectra for compound **5**

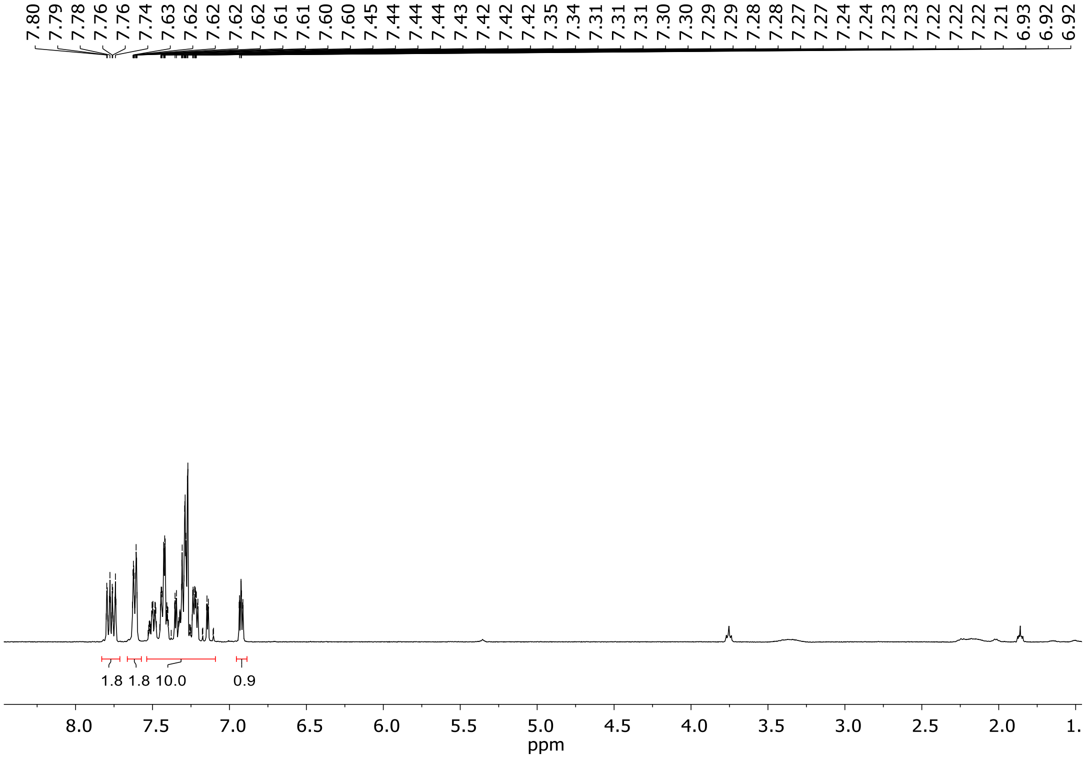

Figure S7: ^{1}H NMR ($CDCl_3$, 400 MHz).

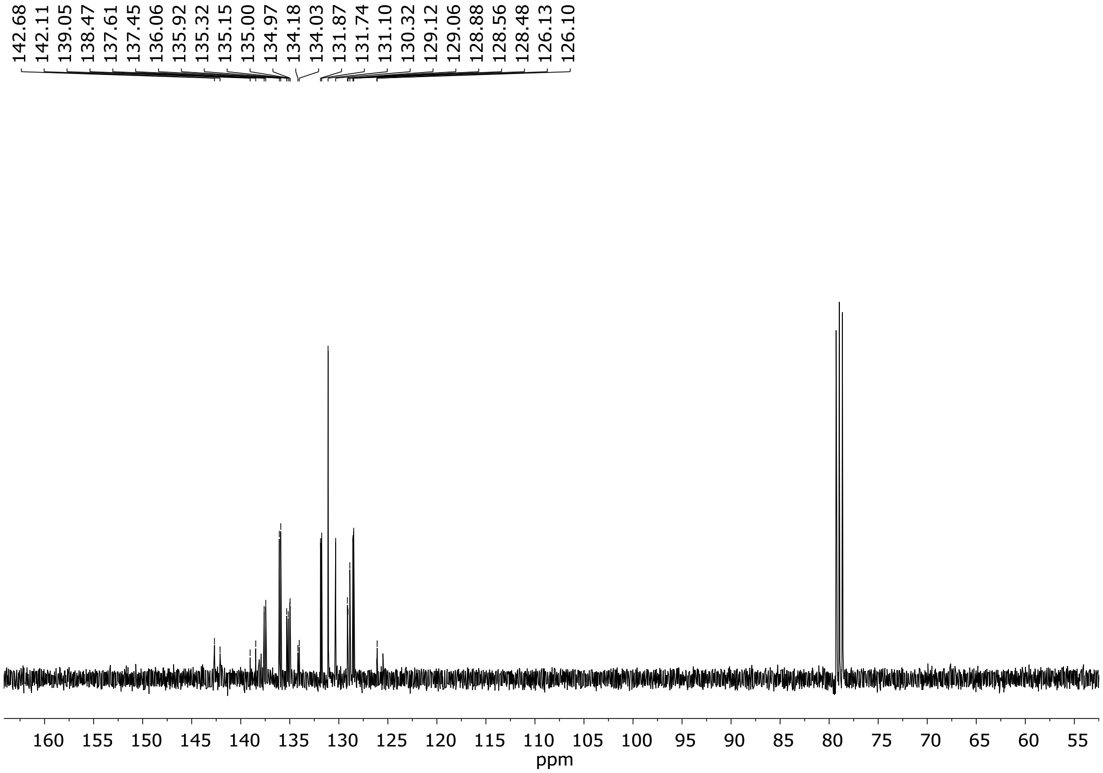

Figure S8: ^{13}C NMR ($CDCl_3$, 101 MHz).

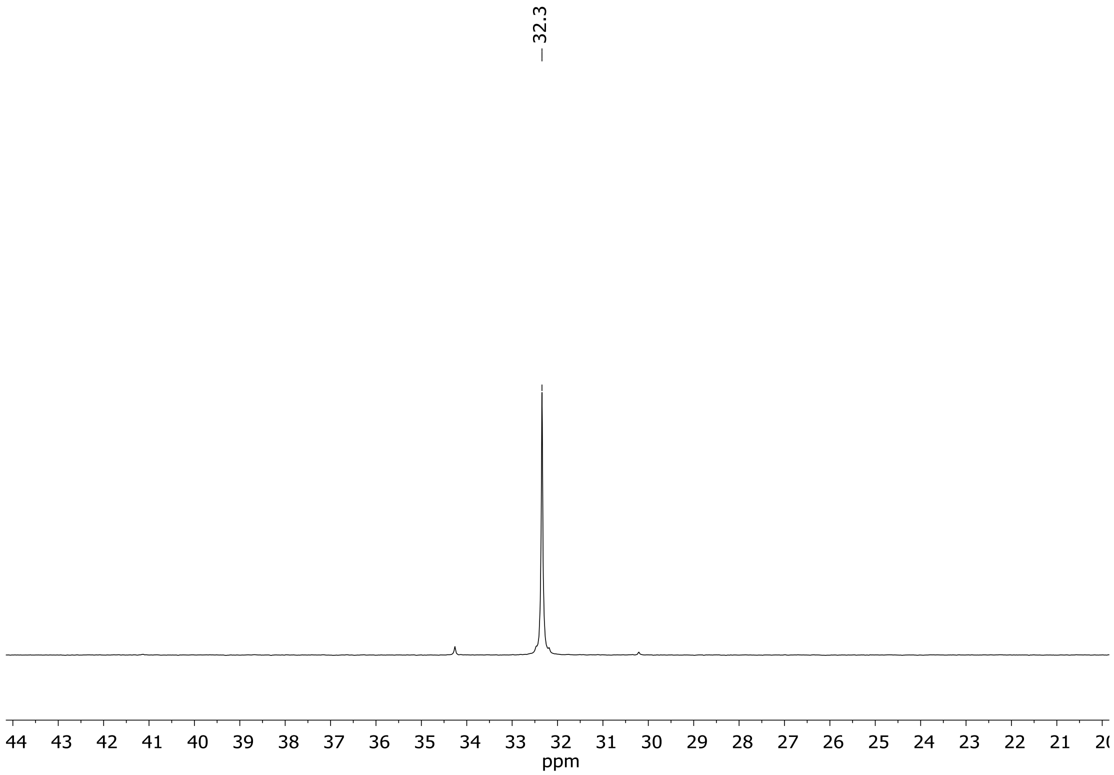

Figure S9: $^{31}P\{^1H\}$ NMR ($CDCl_3$, 202 MHz).

Table S1. ^{1}H DOSY-ECC-MW estimation of $[Cu\mathbf{2}(MeCN)]^+$ (**3**) in CD_2Cl_2 at 25 °C. Cyclopentane was used as internal reference with $\log_{D_{ref,fix}}$(Cyclopentane)[1] = –8.6277. The accuracy of the ECC^{CD2Cl2} (DSE) is in the range of $MW_{dif} \leq \pm 3\%$ and of ECC^{CD2Cl2} (Merge) in the range of $MW_{dif} \leq \pm 5\%$. Hypothetical aggregates are $[Cu\mathbf{2}_m(MeCN)_m]$ with m = 1-2.

**^{1}H DOSY**				**25 °C**
**D_x [m^2/s]**	1.08E-5	**Aggregate**	**MW_{calc} [g/mol]**	**MW_{dif} [%]**
**$\log D_x$**	-4.967	$[Cu\mathbf{2}(MeCN)]^+$	422	3 (DSE)
**$\log D_{x,norm}$**	-8.967			-5 (Merge)
**D_{ref} (Cyclopentane) [m^2/s]**	2.82E-5			-16 (CS)
**$\log D_{ref}$ (Cyclopentane)**	-4.550	$[Cu\mathbf{2}_2(MeCN)_2]^+$	782	91 (DSE)
**MW_{det} [g/mol] (DSE)**	410			75 (Merge)
**MW_{det} [g/mol] (Merge)**	446			55 (CS)
**MW_{det} [g/mol] (CS)**	504			

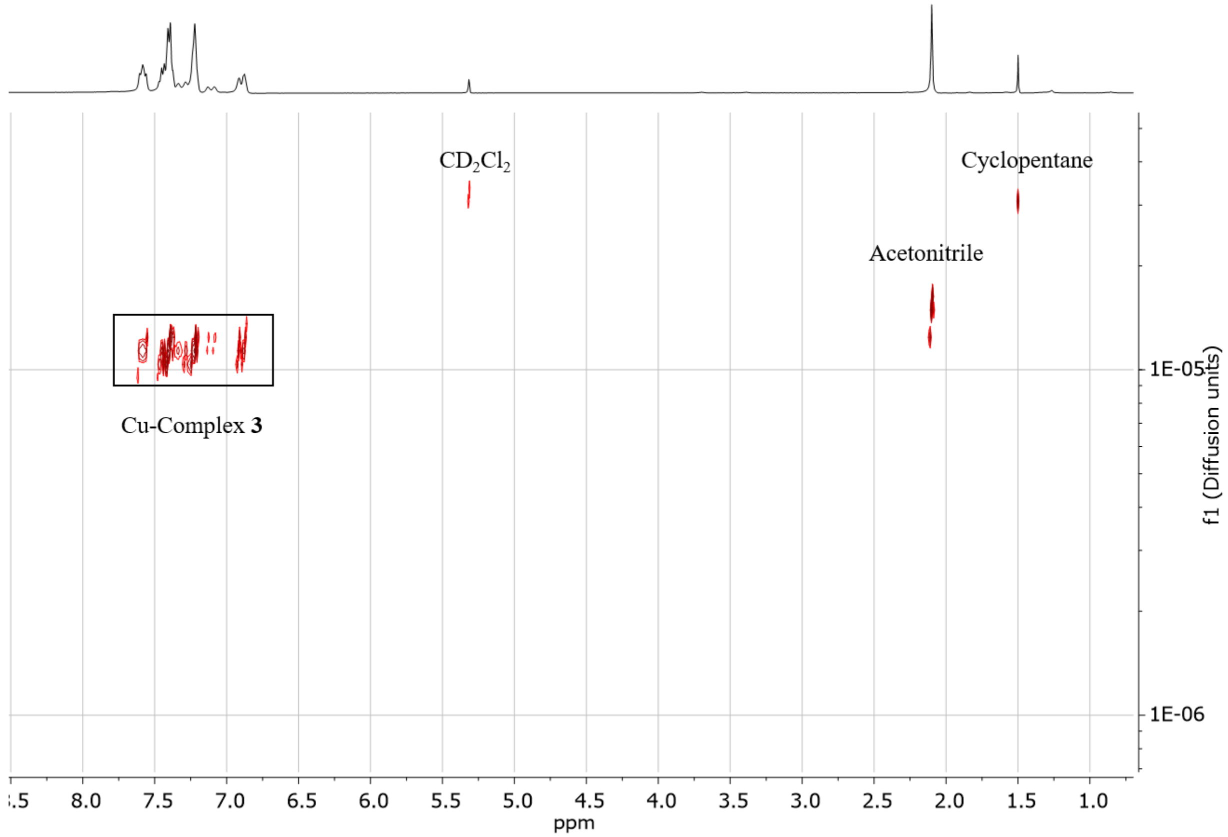

Figure S10: 1H DOSY spectrum of **3** in $CDCl_3$. Internal reference: Cyclopentane.

Table S2. ^{1}H DOSY-ECC-MW estimation of [Ag**2**]$^{+}$ (**4**) in $CDCl_3$ at 25 °C. Cyclopentane was used as internal reference with $\log D_{ref,fix}$(Cyclopentane)[1] = –8.6277. The accuracy of the ECC^{CDCl3} (DSE) is in the range of MW_{dif} ≤ ±13% and of ECC^{CDCl3}(Merge) in the range of MW_{dif} ≤ ±2%. Hypothetical aggregates are [Ag**2**$_m$] with m = 1-2.

**^{1}H DOSY**				**25 °C**
**D_x [m^2/s]**	1.49E-5	**Aggregate**	**MW_{calc} [g/mol]**	**MW_{dif} [%]**
**$\log D_x$**	-4.827	[Ag**2**]$^{+}$	424	13 (DSE)
**$\log D_{x,norm}$**	-9.062			2 (Merge)
**D_{ref} (Cyclopentane) [m^2/s]**	3.84E-5			-10 (CS)
**$\log D_{ref}$ (Cyclopentane)**	-4.416	[Ag**2**$_2$]$^{+}$	743	99 (DSE)
**MW_{det} [g/mol] (DSE)**	374			79 (Merge)
**MW_{det} [g/mol] (Merge)**	414			57 (CS)
**MW_{det} [g/mol] (CS)**	472			

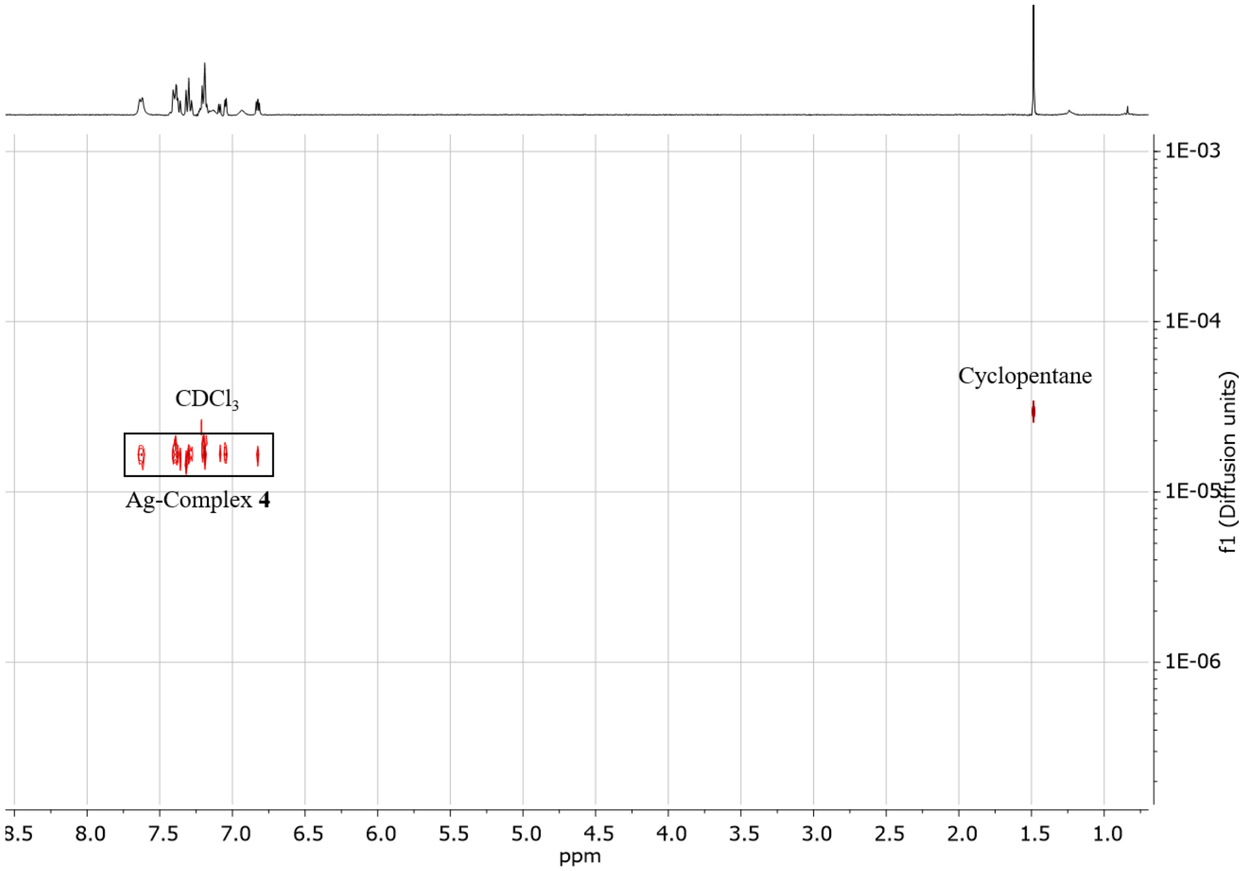

Figure S11: 1H DOSY spectrum of **4** in $CDCl_3$. Internal reference: Cyclopentane.

Table S3. ^{1}H DOSY-ECC-MW estimation of Au**2**Cl (**5**) in $CDCl_3$ at 25 °C. Cyclopentane was used as internal reference with $\log_{D_{ref,fix}}$(Cyclopentane)[1] = –8.6277. The accuracy of the ECC^{CDCl3} (DSE) is in the range of MW_{dif} ≤ ±3% and of ECC^{CDCl3} (Merge) in the range of MW_{dif} ≤ ±0%. Hypothetical aggregates are [Au**2**$_m$]Cl with m = 1-2.

**^{1}H DOSY**				**25 °C**
**D_x [m^2/s]**	1.40E-5	**Aggregate**	**MW_{calc} [g/mol]**	**MW_{dif} [%]**
**$logD_x$**	-4.854	[Au**2**Cl]	551	13 (DSE)
**$logD_{x,norm}$**	-9.128			0 (Merge)
**D_{ref} (Cyclopentane) [m^2/s]**	4.20E-5			-16 (CS)
**$logD_{ref}$ (Cyclopentane)**	-4.377	[Au**2**$_2$]Cl	869	78 (DSE)
**MW_{det} [g/mol] (DSE)**	488			58 (Merge)
**MW_{det} [g/mol] (Merge)**	550			32 (CS)
**MW_{det} [g/mol] (CS)**	658			

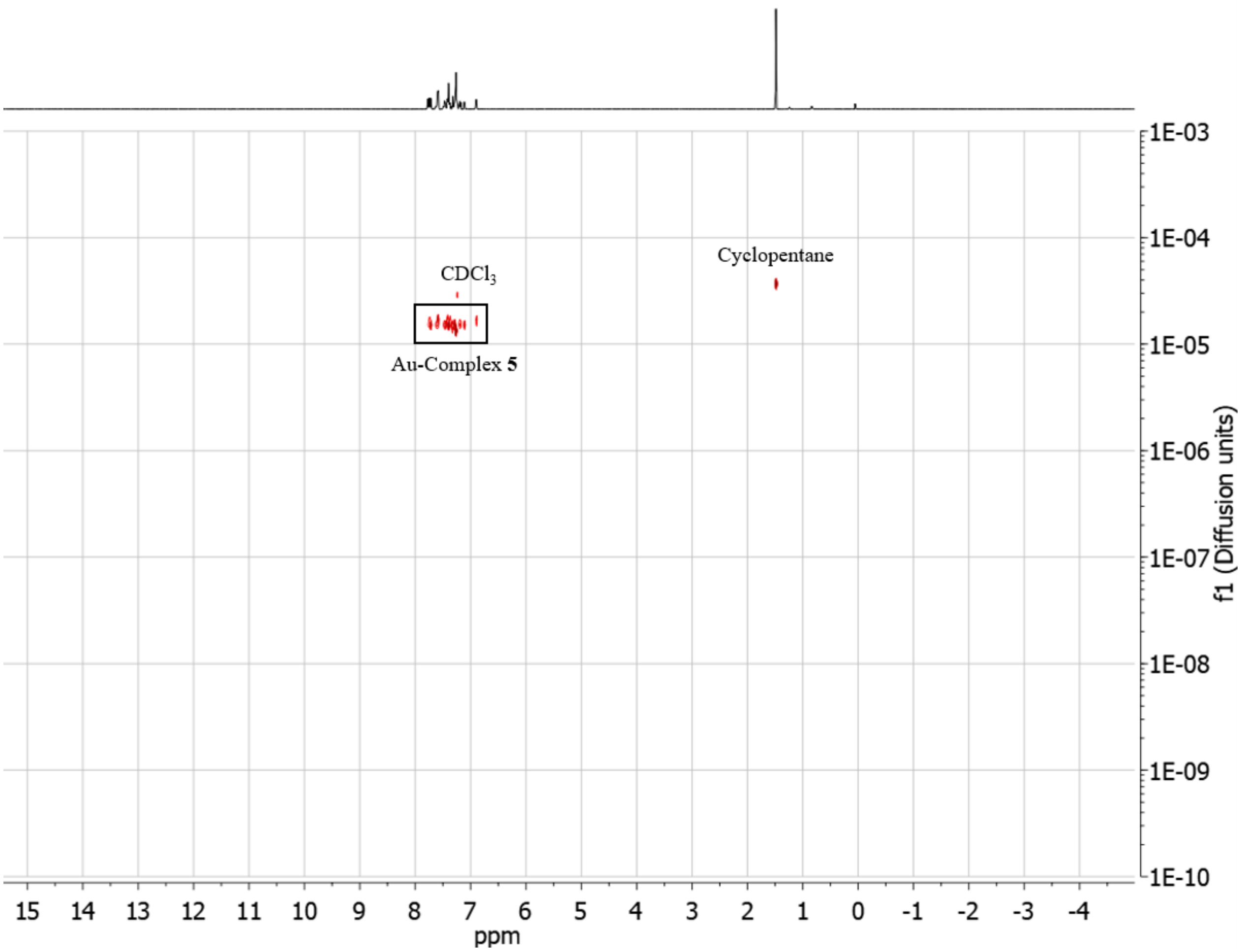

Figure S12: 1H DOSY spectrum of **5** in $CDCl_3$. Internal reference: Cyclopentane.

Table S4. Structure determination and refinement of **3**, **4** and **5**.

Compound reference	**3**	**4**	**5**
Identification code	sv0582	i2836	i2794
Empirical formula	$C_{44}H_{36}BCuF_4N_2P_2S$	$C_{21}H_{17}AgBCl_2F_4PS$	$C_{20}H_{15}AuClPS$
Formula weight	869.16	597.95	550.77
Crystal system	orthorhombic	triclinic	orthorhombic
Space group	*Pbcn*	$P\bar{1}$	*Pbca*
a/Å	10.1811(4)	8.4526(6)	13.2610(19)
b/Å	23.5623(10)	9.7915(8)	14.4928(17)
c/Å	16.8772(6)	13.6796(12)	18.1219(18)
α/°	90	82.674(7)	90
β/°	90	88.693(7)	90
γ/°	90	89.795(6)	90
Volume/Å^3	4048.7(3)	1122.64(16)	3482.8(7)
Z	4	2	8
ρ_{calc}g/cm^3	1.426	1.769	2.101
μ/mm^{-1}	0.775	1.340	8.811
F(000)	1784.0	592.0	2096.0
Crystal size/mm^3	0.11 × 0.1 × 0.09	0.11 × 0.08 × 0.06	0.27 × 0.15 × 0.11
2Θ range for data collection/°	3.458 bis 51.988	3.002 bis 51.526	4.496 bis 51.888
Index ranges	$-12 \leq h \leq 9$, $-29 \leq k \leq 24$, $-20 \leq l \leq 20$	$-8 \leq h \leq 10$, $-11 \leq k \leq 11$, $-14 \leq l \leq 16$	$-16 \leq h \leq 15$, $-17 \leq k \leq 11$, $-20 \leq l \leq 22$
Reflections collected	19577	7900	8684
Independent reflections	3968 [R_{int} = 0.0304, R_{sigma} = 0.0282]	4222 [R_{int} = 0.0273, R_{sigma} = 0.0278]	3255 [R_{int} = 0.0601, R_{sigma} = 0.0491]
Data/restraints/parameters	3968/51/255	4222/0/280	3255/368/343
Goodness-of-fit on F^2	1.082	1.054	1.0503
Final R indexes [I>=2σ (I)]	R_1 = 0.0727,	R_1 = 0.0508,	R_1 = 0.0474,
Final R indexes [all data]	R_1 = 0.0920,	R_1 = 0.0574,	R_1 = 0.0524,
Largest diff. peak/hole / e Å^{-3}	2.07/-1.00	1.61/-1.46	2.40/-1.77
CCDC number	2167961	2167962	2167963

Complex **3** crystallizes in the orthorhombic space group *Pbcn* with four formula units in each unit cell. The thienyl ring is disordered over three positions in a ratio of 60:25:15. The disorder was refined anisotropically.

The silver complex **4**·DCM crystallizes in the triclinic space group $P\bar{1}$ with two formula units in each unit cell. The ratio of the disorder in the thienyl ring is 3:1, and the sulfur atom of the disorder was refined anisotropically.

Compound **5** crystallizes in the orthorhombic space group *Pbca* with eight formula units in each unit cell. The thienyl ring has a disorder in the ratio of 70:30, which was refined anisotropically except for carbon atoms C6A and C14A.

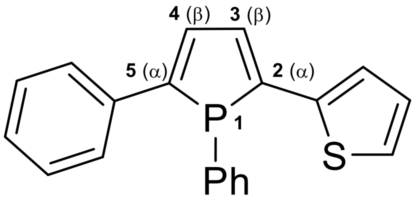

Scheme S1: Ring numbering scheme for the phosphole under investigation, with additional notation of α- and β-positions.

References

[1] Bachmann, S.; Neufeld, R.; Dzemski, M.; Stalke, D. New External Calibration Curves (ECCs) for the Estimation of Molecular Weights in Various Common NMR Solvents. *Chem. Eur. J.* **2016**, *22*, 8462–8465.
